# A Mixed Methods Study of Perceptions of Mental Illness and Self-Disclosure of Mental Illness Among Medical Learners

**DOI:** 10.5334/pme.1152

**Published:** 2024-06-05

**Authors:** Aliya Kassam, Benedicta Antepim, Javeed Sukhera

**Affiliations:** 1 Department of Community Health Sciences, Cumming School of Medicine, University of Calgary, Canada; 2 Department of Community Health Sciences and research associate in the Office of Postgraduate Medical Education in the Office of Postgraduate Medical Education, Cumming School of Medicine, University of Calgary, Canada; 3 Hartford Hospital and the Institute of Living and an Associate Clinical Professor of Psychiatry at Yale School of Medicine, Connecticut, USA

## Abstract

**Introduction:**

Mental illness stigma remains rooted within medical education and healthcare. We sought to measure perceptions toward mental illness and explore perceptions of self-disclosure of mental illness in medical learners.

**Method:**

In a mixed-methods, sequential design, authors recruited medical learners from across Canada. Quantitative data included the Opening Minds Scale for Healthcare providers (OMS-HC), the Self Stigma of Mental Illness Scale (SSMIS), and a wellbeing measure. Qualitative data included semi-structured interviews, which were collected and analyzed using a phenomenological approach.

**Results:**

N = 125 medical learners (n = 67 medical students, n = 58 resident physicians) responded to our survey, and N = 13 participants who identified as having a mental illness participated in interviews (n = 10 medical students, n = 3 resident physicians). OMS-HC scores showed resident physicians had more negative attitudes towards mental illness and disclosure (47.7 vs. 44.3, *P* = 0.02). Self-disclosure was modulated by the degree of intersectional vulnerability of the learner’s identity. When looking at self-disclosure, people who identified as men had more negative attitudes than people who identified as women (17.8 vs 16.1, *P* = 0.01) on the OMS-HC. Racially minoritized learners scored higher on self-stigma on the SSMIS (Geometric mean: 11.0 vs 8.8, *P* = 0.03). Interview data suggested that disclosure was fraught with tensions but perceived as having a positive outcome.

**Discussion:**

Mental illness stigma and the individual process of disclosure are complex issues in medical education. Disclosure appeared to become more challenging over time due to the internalization of negative attitudes about mental illness.

Medical education can take place in a highly stressful environment, particularly for individuals with lived experience of mental illness. Despite the higher rates of psychological distress in medical learners compared to the general population of post secondary learners (20.9% vs. 10.5%), many individuals do not seek help for the valid fear that the stigma of these conditions will tarnish their reputation and careers [1,2,3,4]. Previous research on stigma suggests that stigma can be enacted through dynamic processes that include individual, organizational, and societal level influences, [3,5]. The outcomes of unaddressed mental illness among medical learners are potentially disastrous. Any efforts to enhance the holistic wellbeing of medical learners taking an intersectional approach [6] to health (including their mental health) will remain limited unless the role of stigma related to mental illness in the clinical learning environment is addressed in a meaningful way.

Stigma research has been growing in the field of medical education. Goffman defined stigma as “an attribute that is deeply discrediting and that reduces the bearer from a whole and usual person to a tainted, discounted one” and he articulated that stigma can become internalized and contribute to shame and self-devaluation [7]. Goffman’s focus on stigma at the individual level was broadened by Link and Phelan who highlighted that stigma can be more dynamic and related to cognitive, affective, and behavioral components [8]. Stigma as a societal problem includes stereotypes (negative perceptions/attitudes), prejudice, and discrimination [3]. Stigma can be experienced towards others, or towards oneself [5,9]. Individual-level stigma also includes stereotypes, prejudice, and discrimination against an individual or oneself. Stigma to oneself, also known as self-stigma or internalized stigma, can contribute to a loss of self-efficacy and learned helplessness [5,9]. In a recent study of stigma amongst medical learners, stigma against seeking help was counterbalanced by the ideal of heroic disclosure which was perceived as difficult within a system that upholds cultural norms of perfectionism and invulnerability that are further perpetuated by the hidden curriculum [10,11].

Gaining a deeper understanding of how to address stigma in medical education is related to the need for a deeper understanding regarding the process of self-disclosure. Interventions to reduce self-stigma therefore often promote self-disclosure [9]. The concept of self-disclosure is defined as a process through which individuals verbally reveal themselves to others [12]. By sharing one’s experience, self-disclosure helps to bring stigmatized conditions such as mental illness out of the shadows, disconfirms stereotypes, and normalizes help-seeking. However, self-disclosure may place a disproportionate burden of addressing stigma on those who are affected by it [13,14]. Therefore, despite the potential benefits of self-disclosure, revealing oneself may also have the potential to further harm the wellbeing of medical learners, particularly when others do not receive the information well or use the information to harm and discriminate [15,16,17].

Current knowledge on self-disclosure in medical education is limited. Existing literature includes a few studies that highlight the value of faculty self-disclosure [11,18]; and evidence that medical graduates who disclose are less likely to receive a residency interview invitation [19]. While studies have been conducted to explore attitudes on the topic [20,21], these included little to no empirical investigation of the process of learner self-disclosure, including information about how disclosure relates to self-stigma, learners’ social identities, and the barriers or enablers for the process of disclosure within broader social and structural contexts within medical education. A deeper understanding of self-disclosure among medical learners may help advance our understanding of both the manifestations of mental illness stigma in the clinical learning environment and how to address it.

The aim of our study was to explore perceptions of mental illness and self-disclosure of mental illness in medical students and resident physicians. We sought to explore barriers and enablers of self-disclosure related to knowledge, attitudes, and behaviour [3], through a deeper understanding of the perceived outcomes (both positive and negative) of self-disclosing among medical learners. Our overarching research question was, what are the perceptions of medical learners regarding mental illness and self-disclosure of mental illness? We also sought to understand the following sub-questions: (1) What factors enable or constrain self-disclosure of mental illness in medical learners? (2) What are the perceived positive and negative outcomes of self-disclosure of mental illness in medical learners?

## Method

### Approach

Mental illness stigma research is a diverse field with several epistemologies and theoretical and conceptual lenses that have been applied to stigma research. Our approach was grounded in a biopsychosocial model of illness [22] and the position that mental illness and experiences of mental illness contribute to the biological, psychological, and social dimensions of an individual’s identity [6,22]. We felt that a biopsychosocial model aligns with an integrative framework for stigma research posited by Pescosolido and colleagues [22]. Their Framework on Integrating Normative Influences on Stigma (FINIS) starts with Goffman’s definition [7] while acknowledging the individual and social aspects of stigma from Link and Phelan [8] and considering structural aspects of stigma that may reinforce and perpetuate stigmatizing attitudes and behaviors. As an author team, we have conducted previous research through a variety of epistemological positions on stigma research including previously published work from a critical epistemology [10]. However, for this research, we felt that a pragmatic and mixed methods approach to our research question fit an important gap in existing medical education literature. Therefore, we used pragmatism as our methodological approach [23]. Pragmatism assumes that knowledge is created from socially shared experiences and is influenced by our beliefs, habits, and interactions with others and is the philosophical frame for our study [24]. Our desire was to consider validated questionnaires encompassing cognitive, affective and behavioural domains given their influence on each other which is well-documented in the literature [25], while complementing such questionnaires and data with the strengths afforded by qualitative inquiry.

### Design

In our study, we first identified a common problem among medical learners pertaining to the stigma of mental illness through a quantitative survey and then explored perceptions of self-disclosure through qualitative semi-structured interviews. In keeping with Morse’s approach of sequential qualitative research design, the survey acted as a selection tool and background framing for the qualitative research [quan->QUAL] [26]. Our rationale for using a mixed methods design was to explore perceptions of medical learners regarding mental illness stigma and self-disclosure through complementary yet distinct sources of data. This mixing of methodologies is intentional and aligns with the concept of methodological borrowing as described by Varpio and colleagues [27]. This research was approved by the Conjoint Health Research Ethics Board at the University of Calgary (ID:20-1149) and the Office of Human Research Ethics (OHRE) at Western University (ID: 116634).

### Setting and participants

Canada is home to 17 diverse medical schools spanning a wide geography. Each school varies regarding curricular approach and format. The study team had administrative and infrastructure support at two sites in Canada (one in western Canada and one in eastern Canada) where ethics review was sought. Data collection took place across the 17 medical schools. To ensure the highest level of confidentiality and protection of sensitive information we involved an interviewer who was not in medical education and thus had no influence on the participants’ training or career progression (author B.A.).

### Data Collection

Data was collected between August 2020 and August 2021 and analyzed in 2022. Recruitment for the study took place at the respective co-principal investigators’ home institutions through internal communication channels such as listservs. The goal was to recruit participants from other medical schools in Canada by snowball sampling through email as well as through public posts on social media platforms. Snowball sampling takes place when one or more initial participants recruit others to form part of the sample. This method can be used for sensitive topics such as the stigma of mental illness.

#### Phase 1: Quantitative Survey

The inclusion criteria for participation in the quantitative survey was any current medical learner (medical student or resident) who had experience of a mental illness. Quantitative data was collected using the Qualtrics online survey tool [28]. Our survey consisted of questions about demographic variables that would provide insights into intersectional identities including age, gender identity and ethno-racial identity as well as other variables that might impact mental illness stigma such as marital status, location of medical school, length of medical school, type of medical learner, and contact with a person with mental illness. We also included several previously developed and psychometrically tested questionnaires [29,30,31]. Supplemental material shows the validated questionnaires used [29,30,31]. All continuous quantitative data were tested for assumptions of normality and where data were positively skewed, data were log transformed and geometric means were computed. Continuous data were analyzed using independent sample t-tests comparing mean scores of the questionnaire subscales and demographic variables that were dichotomized. Effect sizes in the form of Cohen’s *d* were also computed to determine the extent of relationships between variables.

#### Phase 2: Qualitative Interviews

We included any currently enrolled or employed medical learner (medical student or resident) who had self-disclosed their mental illness to anyone in their sphere of training. Survey participants were asked to complete a form with their contact information if they were interested in being interviewed for the study. In seeking a rich understanding of the factors that influence self-disclosure of mental illness or for medical learners, we chose to inform the qualitative arm of our study with phenomenological inquiry as a methodology. Hermeneutic phenomenology emphasizes the limitations of “bracketing” for the research team [32]. During our analysis we openly reflected on, shared, and attended to subjectivity from personal lived experiences during an iterative process of data collection, reflection, reflexivity, and analysis [33].

We conducted qualitative analysis on both open comments in the survey and throughout the semi-structured interviews. Two investigators (J.S. and B.A.) developed a semi-structured discussion guide based on existing literature on self-disclosure. Questions focused on eliciting narratives of experience. We asked about each participant’s experience of disclosure including predisposing factors, motivations to disclosure, reactions from self, reactions from others, and perceived meaning of disclosure for their future career as a physician and/or educator. We scheduled 90 minute interviews to allow for deeper probing and reflection on participant experiences consistent with a phenomenological approach. Interviews were audio-recorded and transcribed verbatim as well as de-identified before analysis.

Data were analyzed in accordance with the tenets of hermeneutic phenomenological analysis described in the literature, [32,34] which identifies participants’ interpretations and first-order constructs that are subsequently integrated with the researcher’s second order constructs. Stages include immersion, understanding, abstraction, synthesis, illumination, and integration [32]. Both J.S. and B.A. consensus coded the first several transcripts inductively and then J.S. conducted independent coding that was discussed at regular intervals with other members of the team.

### Reflexivity

Our team acknowledged that methodological borrowing requires that scholars be competent in both methodologies [27]. Both co-primary investigators (A.K. and J.S.) have doctorates in health services research and medical education respectively, are full-time academics who are well-versed in both qualitative and quantitative research methods as well as share an interest in mental illness. Author J.S. is also trained as a psychiatrist and A.K. is trained as a psychiatric epidemiologist/health services researcher. They work separately in distinct academic medical centers that teach medical learners. All members of the research team are racialized and have varied lived experiences of stigma, and discrimination.

In conducting this research, both investigators and research team members intended to consistently and iteratively test their own assumptions and the framing of the issue of mental illness stigma and self-disclosure of mental illness based on their own personal and professional experiences. Since the investigators had previous knowledge of mental illness, they had to be aware that if there were findings from a participant that were unexpected or did not fit with preconceived assumptions of ideas, that this was an opportunity to reflect on these previous assumptions. When analyzing interviews, the investigators sought to remain open and non-judgmental. Given that the data is largely a result of the interaction between the investigators and the participants in interviews, the investigators and research team sought ongoing critical awareness of this relation. The investigators have also used reflexive strategies such as acknowledging multiple frameworks of stigma, models of illness and seeking to understand both the barriers and enablers to self disclosure, as well as both positive and negative outcomes of disclosure. The results section has been modeled after similar sequential mixed methods research [35].

## Results

Analysis suggests that mental illness stigma and self disclosure was a complex process in the context of medical education. Self disclosure appeared to become more challenging over time due to the internalization of negative attitudes about mental illness. OMS-HC scores showed resident physicians had more negative attitudes towards mental illness and disclosure. Overall findings also suggest that self-disclosure depended upon the degree of intersectional vulnerability of the learner’s identity and diagnosis. For example, the more intersections a learner’s identity encompassed, the more likely they perceived their self-disclosure of their mental illness or addition would be stigmatized. When looking at self-disclosure, people who identified as men had more negative attitudes than people who identified as women. Racialized learners scored higher on self-stigma. Qualitative data amongst participants with intersectional identities struggled in unique and varied ways not only according to other aspects of their identity such as gender and racialization, but also according to their perception of stigma related to their diagnosis. Overall, findings suggested that the disclosure journey was fraught with tensions, but ultimately lead to positive outcomes.

### Phase 1: Quantitative Survey

Overall, 125 medical learners (n = 67 medical students, n = 58 resident physicians) responded to our survey. Table 1 shows the demographic data of the survey participants. Of the 77 participants that responded to the survey question about disclosure, 36.3% reported having disclosed a mental illness within their learning environment whereas 24.7% have only considered disclosing a mental illness. Due to non-applicable responses and missing data, not all percentage estimates will total 100.

**Table 1:** Demographic Characteristics of Survey Participants.


SOCIO-DEMOGRAPHIC VARIABLE	ESTIMATE

**Mean Age (SD)**	27.2 (3.8)

**Medical Learner % (n)**	

Medical Student	53.6% (67)

Resident	45.6% (57)

**Gender % (n)**	

Woman	72.8% (91)

Man	24.0% (30)

Non-Binary	1.6% (2)

Prefer not to answer	0.8% (1)

**Institution % (n)**	

Eastern Canada	24.8% (31)

Western Canada	74.4% (93)

**Type of Medical School % (n)**	

3-Year Medical School	24.0% (30)

4-Year Medical School	76.0% (95)

**Ethno-racial Identity % (n)**	

White	51.2% (64)

Korean	4.0% (5)

Japanese	0.8% (1)

Other	1.6% (2)

South Asian (e.g., East Indian, Pakistani, Sri Lankan)	12.0% (15)

Chinese	10.4% (13)

Filipino	1.6% (2)

Arab	3.2% (4)

Mixed or Biracial	8.0% (10)

Southeast Asian (e.g., Cambodian, Laotian, Vietnamese)	1.6% (2)

West Asian and Middle Eastern (e.g., Afghan, Iranian, Israeli)	4.8% (6)

Prefer not to answer	0.8% (1)

**Marital Status % (n)**	

Never legally married (Single)	76.8% (96)

Legally married, not separated	20.0% (25)

Divorced	2.4% (3)

**Has close contact with someone who has mental illness (n)**	

Yes	66.4% (83)

No	12.0% (15)


All continuous variables were positively skewed except for the WHO-5 wellbeing score, Attitudes towards Disclosure subscale score and the total score of the Opening Minds Scale for Healthcare Providers (OMS-HC) shown in Table 2. Medium effect sizes (0.3–0.5) were found with racialized learners having more stigmatizing attitudes overall as well as with respect to disclosure. Medium effect sizes were also found with learners identifying as women having lower stigmatizing attitudes with respect to disclosure. Large effect sizes (0.8 and above) were found with learners who had close contact with a person with mental illness having lower stigmatizing attitudes overall as well as with respect to disclosure. Overall scores of the WHO-5 showed poor wellbeing across the participants with a mean total score of 12.5 (95% CI 11.5–13.5). Medical learners who were married and not separated had statistically significant higher wellbeing scores than those who were single (14.3 vs. 12.2, *P* = 0.03). Across all other stratified groups of medical learners, wellbeing scores prompted a signal to screen for depression given that participants scored 13 or below.

**Table 2:** Non-skewed Measures of Wellbeing, Attitudes Towards Mental Illness (MI) and Attitudes Towards Disclosure of MI.


	MEASURE		

	WHO-5	OMS-HC – TOTAL SCORE	OMS-HC- DISCLOSURE /HELP-SEEKING SUBSCALE

DEMOGRAPHIC VARIABLE	MEAN (SD, 95% CI)	MEAN (SD, 95% CI)	MEAN (SD, 95% CI)

Three Year Medical School	12.6 (5.0, 11.5–13.8)	46.1 (7.3, 44.4–47.8)	16.5 (3.3, 15.7–17.3)

Four Year Medical School	12.3 (4.9, 10.0–14.5)	45.4 (6.0, 42.4–48.4)	16.4 (3.3, 14.8–17.9)

Cohen’s *d* Effect Size	0.08	0.09	0.05

Eastern Canada	12.7 (5.1, 11.5–13.8)	44.9 (5.9, 42.1–47.7)	16.1 (3.2, 14.7–17.6)

Western Canada	12.5 (4.9, 10.0–14.5)	46.2 (7.3, 44.5–48.0)	16.6 (3.3, 15.8–17.4)

Cohen’s *d* Effect Size	0.03	0.19	0.14

Medical Student	12.7 (5.5, 11.2–14.3)	**44.3 (6.6, 42.3–46.2)** ^b^	16.1(3.1, 15.2–17.0)

Resident	12.4 (4.3, 11.1–13.6)	**47.7 (7.1, 45.6–50.0)** ^b^	16.9 (3.5, 15.8–17.9)

Cohen’s *d* Effect Size	0.08	0.50	0.22

Identifies as Woman^a^	13.0 (4.8, 11.8–14.1)	45.3 (7.0, 43.6–46.9)	**16.1 (3.5, 15.2–16.9)** ^b^

Identifies as Man^a^	11.3 (5.3, 9.0–13.6)	48.1 (6.9, 45.1–51.2)	**17.8 (2.4, 16.8–18.8)** ^b^

Cohen’s *d* Effect Size	0.33	0.41	0.53

Non-white identifying	12.5 (5.4, 10.7–14.2)	47.3 (7.2, 44.9–50.0)	16.9 (3.4, 15.9–18.1)

White identifying	12.6 (4.6, 11.4–13.9)	44.9 (6.8, 43.0–46.8)	16.2 (3.2, 15.2–17.0)

Cohen’s *d* Effect Size	0.02	0.34	0.25

Never legally married	**12.2 (5.2, 11.1–13.4)** ^b^	46.1(7.1, 44.4–47.7)	16.6 (3.3, 15.9–17.4)

Legally married, not separated	**14.3 (3.6, 12.7–16)** ^b^	44.9 (6.2, 41.9–47.8)	16.0 (3.5, 14.3–17.7)

Cohen’s *d* Effect Size	0.42	0.17	0.18

Close contact with person with MI	12.8 (5.0, 11.7–13.8)	45.2 (6.9, 43.6–46.7)	16.1 (3.2, 15.3–16.7)

No close contact with person with MI	11.3 (4.7, 8.4–14.2)	51.2 (5.8, 47.4–54.9)	19.4 (2.1, 18.0–20.8)

Cohen’s *d* Effect Size	0.29	0.88	1.07


^a^We had categories for learners identifying as non-binary as well as for self-description of gender identity however the cell-sizes were less than n = 5 and are not reported here. ^b^Estimates in **bold** type font are statistically significant using independent samples t-tests (WHO-5 score at *P* = 0.03 (one-tailed), OMS-HC score at *P* = 0.02 (two-tailed) and OMS-HC Subscale score at *P* = 0.01 (two-tailed)).

On the OMS-HC, resident physicians had more negative attitudes towards people with mental illness and disclosing a mental illness (47.7 vs. 44.3, *P* = 0.02). When looking at the Attitudes towards Disclosure subscale of the OMS-HC, people who identified as men had more negative attitudes than people who identified as women (17.8 vs 16.1, *P* = .01).

Regarding the Self-Stigma of Mental Illness (SSMIS) scale, people who identified as racially minoritized learners scored higher in the applying stigma of mental illness to oneself (Apply) subscale when compared to learners who identified as white (Geometric mean: 11.0 vs 8.8, *P* = 0.03).

Table 3 shows the demographic characteristics of the interview participants.

**Table 3:** Demographic Characteristics of Interview Participants^a^


LEARNER TYPE	INSTITUTION	AGE	GENDER IDENTITY	MARITAL STATUS	LIVING WITH COMMON LAW	ETHNO-RACIAL IDENTITY

Medical student	Western Canada	22	Woman	Single	No	Chinese

Medical student	Western Canada	25	Man	Single	Yes	White

Medical student	Western Canada	24	Man	Single	No	White

Medical student	Eastern Canada	24	Woman	Single	No	South Asian

Medical student	Eastern Canada	25	Woman	Single	No	West Asian or Middle Eastern

Medical student	Eastern Canada	23	Woman	Single	No	Southeast Asian

Medical student	Eastern Canada	26	Woman	Single	Yes	White

Resident	Western Canada	29	Woman	Single	No	White

Resident	Western Canada	29	Woman	Married	No	White


^a^The additional n = 5 medical students and n = 1 resident in this sample did not wish to disclose their demographic information.

### Phase 2: Qualitative Interviews and Survey Comments

#### Barriers and Enablers to Self-Disclosure

The most prominent barriers to disclosure included fear and stigma. Fear was perceived as fear of judgment from peers, and fear of retribution or career outcomes from those with more structural power such as preceptors or organizations themselves. Among medical student participants, many shared their fears of appearing as though they were substandard or not up to par as learners or future physicians. For example, one medical student participant described a fear “that I’m going to be a bad doctor somehow,” and realized that this was due to their internalization of views of what would be considered a good doctor (M1). Among resident participants, however, there was more a fear of negative outcomes for their career than a fear of judgment. Several resident participants cited fear of future licensure applications including a survey comment that there is:

“Not just a fear of retribution for disclosing but knowing this happens from watching classmates disclose their struggles…watching residency programs force residents to take mental health leave for either real mental health concerns or as a power tool to intimidate residents into doing what the program wants or the residents permanently has a record of taking mental health leave. Taking leave from residency, or being diagnosed with a mental health condition, or being on/ having ever received treatment can affect future licensing and license applications and it can lead to mental health exclusions in future critical illness or disability insurance.” Survey Participant 01

Another resident participant stated,

“And so, I think I had a lot of – it’s not really fear of retribution, but just really recognizing that requesting any sort of accommodations in work would really negatively affect my learning experience in residency. And affect my job prospects down the road, because I think it would be very quickly construed as laziness or just maybe the inability to kind of unable to hack it, which is a very common trope.” (R2)

Both quotes highlight participants’ fear. The first notes the visceral effects of witnessing perceived discrimination of peers for mental health challenges and the second notes how such fear affects not only disclosure, but also help-seeking behavior. These align with the phase 1 results suggesting that resident physicians demonstrate more negative attitudes towards people with mental illness and disclosing a mental illness than do medical students.

There were social and individual enablers of disclosure. The most prominent enabler was when learners had a preceptor that demonstrated genuine and empathic understanding, was non-judgmental, and preceptors who role modeled vulnerability. Supportive peers were also considered an enabler, along with having reassurance of anonymity and inclusive and transparent policies that would help learners know what would happen if they disclosed. Overall, participants noted that access to resources and an open and safe learning environment where disclosure is normalized serves as an enabler. At an individual level, participants noted that before they could disclose to others, they needed to give themselves permission to self-disclose. The concept of ‘permission to self’ included overcoming an individual’s internalized fears of being weak, less-than, or somehow not worthy of their place in the profession. Participants described working towards self-acceptance and perceiving a sense of agency or self-efficacy before feeling prepared to disclose to others. In contrast, when it came to enablers from one’s environment, an individual’s perceived comfort, perceived support, and perceived closeness to their peers were prominent enablers. Participants tended to disclose more to peers who were like them, however, some disclosed to preceptors and others to their organization’s wellness or wellbeing offices. There were also a few instances of accidental disclosure which occurred when participants sought accommodations and disclosed to acquire support that they perceived as necessary from their institution.

Most participants described a precipitant to their disclosure. The precipitant varied whether it was among peers, to their preceptor, or to their institution. Across all situations, there was a need for some type of facilitation or mediation before disclosure was elicited. In some circumstances it was a supportive preceptor who facilitated disclosure through gentle or non-judgmental questioning, in others it could be peers who made the discloser comfortable by demonstrating support, openness, and trust. Ultimately, motivations to disclose included a desire to support learning or gain accommodation, but also to help others or to role model vulnerability.

#### Variation in the Disclosure Journey

Overall, we found that there was tremendous variation by context, perceived identity, and diagnosis. Most individuals described unique and individual disclosure journeys and experiences. Among the participants, several described how their diagnosis or diagnoses had a unique influence on how they experienced disclosure. For example, one participant stated that they were not sensitive about their ADHD diagnosis but were concerned about “stigma around a diagnosis of bipolar” (M5), while another disclosed their bipolar disorder but were hesitant to disclose their obsessive-compulsive disorder diagnosis (M8).

The degree that an individual’s self-perception of being different from others also varied. For example, one medical student stated that they felt like an “outsider” in the system and that their experience as an outsider influences how they feel about themselves and their discomfort with disclosure. (M7). Similarly, another participant shared that men have “their own particular challenges” with disclosure that are unique from other gender identities (M8), and one described how they feel “disclosures about my depression is kind of akin to disclosures about my sexuality…they are definitely interrelated.” (M2). These results align with the phase 1 findings that highlight that racially minoritized learners scored higher in self-stigma of mental illness.

#### Disclosure, Double Standards, and Internalization of Self-Stigmatizing Beliefs

A finding across all codes, groups, and participants is related to how disclosure was understood and experienced uniquely in the context of medical training. Participants described a sense of hypocrisy, double standards, or duplicity. Several noted that application processes seemed to seek students who were well rounded and that narratives of adversity were often anticipated, yet their experiences in medical training were discordant to their expectations and their conceptualization of what would make a good physician. One participant described cultural norms in medicine that might be reinforced within the hidden curriculum by stating,

“But, this happens so much in medicine. We, like, talk about illnesses as if no one in the room could possibly have it. Like we talk about diabetes with this assumption, like, no one has diabetes. We talk about hepatitis as if no one has or has ever gotten hepatitis. We talk about OCD in this case and compulsions as if no one has compulsions in the room.” (M8)

The distress shared by participants related to their perception of standards is also reflected in phase 1 results that demonstrate poor overall wellbeing.

#### Outcomes of Disclosure

Generally, participants described feeling positive after disclosure. They felt better and more confident about themselves. They also felt a sense of validation from their peers; closeness, and camaraderie that made them feel validated after their disclosure. Participants also noted that they felt that disclosing would help make them a better physician, have more empathy for patients, and be a better educator.

Table 4 provides additional quotes from our qualitative findings.

**Table 4:** Representative Quotes from Qualitative Findings.


BARRIERS	ENABLERS	OUTCOMES	DOUBLE STANDARDS

*Fear of judgment*	*Permission to self*	*Better about self*	*Internalized Stigma*

“Does this make me look like I’m weak or not good; that I’m going to be a bad doctor some how…I realized those are totally untrue, but I think that was just my internal monologue.” – M1	“Disclosing is part of the whole process of acknowledging that happened to you and working to change your thinking.” – M9	“Being able to disclose has taught me that I’ve accepted it; and the more I’m able to disclose that and share that the more accepting I am of myself, and you know…those struggles.” – M2	“My own self stigma is just the self doubt part that is more of an internalized thing…an internalized fear…and just my own performance being affected.” – M6

*Fear of retribution*	*Peers*	*Better doctor*	*Hypocrisy*

“It was just so embarrassing, so I didn’t want people to know if they didn’t have to know or if they weren’t going to help me. Just because I knew that would kind of change dynamics. And I didn’t want anything on a permanent record either.” – R1	“It was the comfort of ‘I’m not in a room with strangers anymore, I’m in a room with people who know me, and I know them.’” – M6	“I think it (disclosure) makes me able to ask more insightful questions and show some understanding that helps me build rapport with people quickly.” – M12	“There’s learner wellness and wellbeing …its almost a buzz word I find for the schools, but I don’t really see it put into action.” – M10

*Culture of medicine*	*Preceptors*	*Better teacher*	*Culture of Medicine*

“When I think of students in medical school, it just feels like everyone is doing so well and everyone’s so smart and it seems like no one’s really struggling or having problems with anything.” – M4	“She was just super warm…so open and curious…she asked a few questions, and it felt like it was going to be okay to say it.” – R3	“I would love to teach in the future… and hope that I would be able to create an environment…that would make someone with similar difficulties…feel very comfortable and open to share that with me.” – R3	“We are taught that empathy is the cornerstone of medicine…and then on the other hand we have the way that we treat each other, which is traditionally a very intimidating, hierarchical approach to teaching.” – R1


## Discussion

Our exploration of mental illness self-disclosure among medical learners highlights the complexity of individual learners’ disclosure journey. Our findings suggest that medical learners internalize pervasive norms regarding mental illness, disclosure, vulnerability, and what constitutes a competent or effective physician. Disclosure experiences varied based on individuals’ intersectional identity and perceived diagnosis and appeared to become more difficult over time. However, disclosure was ultimately perceived as having positive outcomes for individuals and their beliefs about themselves and place in medicine.

Our findings suggests that despite being positive and complementary to participants’ professional identity formation, disclosure was difficult, fraught with tension, and varied according to their developmental stage in training. Our findings also support the relevance of employing an intersectional approach when assessing medical learner self-disclosure decision-making and wellbeing [13]. We learned that the participants scored within a range of poor overall wellbeing, and our findings complement other research that suggests that medical training is a unique risk factor for poor wellbeing, which is further compounded by barriers to self-disclosure [1,2,36,37].

Our participants confirmed the persistent double standards and perceived hypocrisy in medical education and that these double standards and fear of disclosure appears to worsen over time [38]. For example, in our study OMS-HC scores and qualitative findings demonstrated that fear of judgment among medical students becomes fear of retribution or adverse career outcomes for residents. This is supported by the literature that shows any positive changes that may occur through best intended efforts of stigma reducing contact-based education with patients with mental illness early on in undergraduate training tend to decay over time as learners progress through the system [16,39]. Yet, in a recent discourse analysis, participants also shared that discourses of vulnerability were in direct contrast to discourses of perfection, and that emotional suppression is taught both explicitly and implicitly to medical learners. Perfection and emotional suppression aligns with Goffman’s theory of “front” behaviors that conceal undesirable behaviours that are subject to judgment by others [40,10]. Our findings therefore suggest that conventional approaches to support stigma reduction and foster disclosure may not be as effective in the context of medical education.

The system of medical education may itself be a barrier to self-disclosure thereby perpetuating a culture of perfectionism. Since we found that self-disclosure could be perceived as positive once it has happened based on learners’ perspectives, our study suggests that the existing approaches that might foster disclosure must first address mental illness stigma and the longstanding norms of perfectionism. Structural changes and accountability through regulatory and accreditation standards may be necessary. For example, accrediting bodies may want to consider how medical schools address stigma and normalize self-disclosure by preceptors and learners alike, which can be an active ingredient in learner self-disclosure [3,16,30]. There is a need for policy development around self-disclosure as a competency for wellbeing. Furthermore, policies will need to reflect how best to foster self-disclosure in medical education while determining the extent to which self-disclosure be confidential – for example with respect to accommodations during training.

Our work also complements a growing body of literature in medical education on the topic of disclosure related to marginalized and minoritized identities. For example, research on disability disclosure highlights that structures of disability disclosure do not align with recommendations from regulatory bodies and that structural barriers are pervasive [17,41]. We join our colleagues who have found that stigma, bias, and a lack of a clear institutional process for disclosure limit access to accommodations [42], and therefore greater transparency and adherence to best practices are needed throughout the continuum of medical education.

The process of facilitating disclosure was varied but ultimately shaped by all actors and influences, highlighting the importance of everyone’s role in medical learners’ sphere of influence in addressing stigma [11]. Some participants disclosed to peers, others to preceptors, and some to their institution itself. The key enabler included perceptions regarding support and safety. Social support was a prominent influence as participants who were married reported higher wellbeing scores as well as qualitative findings pertaining to support. This form of social support was highlighted as coming from peers, preceptors, and family members alike.

Lastly, given the intersectional aspects of how medical learners perceive their identity and how such factors influence disclosure, we recommend that medical schools and residency programs consider the importance of disaggregated socio-demographic data about learners’ intersectionality when planning and implementing wellness initiatives and interventions. For example, people who identify as men may have more negative attitudes towards self-disclosure due to societal norms and racialized learners may be more likely to experience self-stigma based on their own cultural norms about mental illness and potential fear of double discrimination [43,44], which ultimately has downstream impacts for health equity. Our findings should help inform any interventions to reduce stigma by ensuring that other aspects of social identity are addressed, named, and space is created for thoughtful conversations.

Our study had several limitations. First, we collected qualitative data solely on the disclosure of mental illness rather than a holistic perspective of learner wellbeing [37] taking into consideration other aspects of health such as financial stability, physical illness etc. which can have an impact on mental health as well as overall wellbeing. Second, measuring stigma related to mental illness may always have the risk of social desirability bias, which may skew the data. Given that the data we found on several of our measures was positively skewed, we are unable to ascertain whether participant attitudes were positive or whether participants wanted to appear socially desirable due to fear of retribution. Third, our sample size for the quantitative arm of the study was small and the quantitative results should be interpreted with caution. We recognize the limitations of recruiting individuals for a study with a sensitive and challenging topic such as self-disclosure. That said, there is enough evidence given the synthesis of the quantitative and qualitative results of a signal in the noise suggesting further research into intersectional identities in medical learners and the role they play in mental illness stigma and self-disclosure of mental illness. Fourth, we used a long battery of questionnaires that may have imposed response burden for participants and may have contributed to a smaller sample size of survey participants and missing data. Last, it may be seen as a limitation by others that we used the term “mental illness” in a study on stigma and discrimination, which may be seen as perpetuating ableist beliefs.

Future research will need to address the impact of identity markers on medical learner experiences of training and the impact of the learning environment on self-disclosure and wellbeing, for example how the disclosure occurs i.e., peer-to-peer vs. to individuals of power vs. unintended disclosures for accommodation.). Further exploration is also warranted on how best to tackle the structural and cultural barriers in medical education to foster self-disclosure in an ethical manner as well as the role of mentorship in facilitating disclosure.

## Conclusions

The process of disclosing one’s mental illness among medical learners is complex. Self disclosure appeared to become more challenging over time due to the internalization of negative attitudes about mental illness. Intersectional vulnerability in medical learners warrants further consideration because it may modulate disclosure. Fear of disclosure is an important factor shaped by the learning environment that has implications for learner wellbeing, patient care, and health equity.

## Additional File

The additional file for this article can be found as follows:

10.5334/pme.1152.s1 Supplementary Material. Validated Questionnaires.

## Previous Presentations

Kassam A, Antepim B, & Sukhera J. (2023). Exploring perceptions of self-disclosure in medical education. *Association for Medical Education in Europe*, Glasgow, Scotland, August 2023.

Kassam A, Antepim B, & Sukhera J. (2023). Exploring perceptions of self-disclosure in medical education. *International Congress on Academic Medicine*, Quebec City, Canada, April 13–18, 2023.

Kassam A, Antepim B, & Sukhera J. (2022). Exploring Perceptions of Self-Disclosure in Medical Education. *Transform MedEd Conference*, London, England, November 11–12, 2022.
